# AMPAkines potentiate the corticostriatal pathway to reduce acute and chronic pain

**DOI:** 10.1186/s13041-021-00757-y

**Published:** 2021-03-02

**Authors:** Fei Zeng, Qiaosheng Zhang, Yaling Liu, Guanghao Sun, Anna Li, Robert S. Talay, Jing Wang

**Affiliations:** 1 Department of Pain, The First Affiliated Hospital, Nanchang University, Nanchang, Jiangxi People’s Republic of China; 2 Department of Anesthesiology, Perioperative Care and Pain Medicine, New York University School of Medicine, New York, NY USA; 3 Department of Neuroscience & Physiology, New York University School of Medicine, New York, NY USA

**Keywords:** Prelimbic cortex, Nucleus accumbens, AMPAkine, CX 546, Pain

## Abstract

The corticostriatal circuit plays an important role in the regulation of reward- and aversion-types of behaviors. Specifically, the projection from the prelimbic cortex (PL) to the nucleus accumbens (NAc) has been shown to regulate sensory and affective aspects of pain in a number of rodent models. Previous studies have shown that enhancement of glutamate signaling through the NAc by AMPAkines, a class of agents that specifically potentiate the function of α-amino-3-hydroxy-5-methyl-4-isoxazolepropionic acid (AMPA) receptors, reduces acute and persistent pain. However, it is not known whether postsynaptic potentiation of the NAc with these agents can achieve the full anti-nociceptive effects of PL activation. Here we compared the impact of AMPAkine treatment in the NAc with optogenetic activation of the PL on pain behaviors in rats. We found that not only does AMPAkine treatment partially reconstitute the PL inhibition of sensory withdrawals, it fully occludes the effect of the PL on reducing the aversive component of pain. These results indicate that the NAc is likely one of the key targets for the PL, especially in the regulation of pain aversion. Furthermore, our results lend support for neuromodulation or pharmacological activation of the corticostriatal circuit as an important analgesic approach.

## Introduction

Acute pain is an important sensory event that protects us from physical harm and environmental danger. Chronic pain, however, affects one in four adults worldwide and can lead to debilitation and functional impairment. A better understanding of the endogenous pain regulatory pathways can unlock new treatments for both severe acute pain and more importantly for chronic pain.

The prefrontal cortex (PFC) is a highly evolved structure in the brain that provides top-down regulation of a number of sensory and affective behaviors [1–3]. Previous studies have shown that this region has the capacity to regulate both sensory and affective components of pain [4–8]. Recent studies have shown that activation of the prelimbic region of the PFC (PL) in rodents can reduce both sensory withdrawals and aversive responses to pain [6, 7, 9]. The rodent PL shares a strong functional homology with the dorsolateral PFC in primates, a region that is known to be involved in pain processing and regulation [10–17].

The PFC has widespread connections in the brain. It projects to the periaqueductal gray (PAG) to provide outputs to the rostral ventral medulla (RVM) to form a well-known descending inhibitory circuit [18–22]. The PFC also projects to the nucleus accumbens (NAc), a key component in the reward pathway [23–28]. The NAc is known to play an active role in the regulation of pain behaviors [15, 28–35]. Recent studies have shown that the projection from the PL to the NAc in rodents inhibits both sensory and aversive components of pain [6, 7, 9]. Meanwhile, in humans, altered connectivity between the dorsolateral PFC and the NAc has been shown to be an important feature of chronic pain [12, 36].

AMPAkines are a class of compounds that bind to an allosteric site on the α-Amino-3-hydroxy-5-methyl-4-isoxazolepropionic acid (AMPA) receptor to prevent receptor deactivation [37, 38]. Hence, these drugs potentiate the function of already activated AMPA receptors to increase central glutamate signaling in a use-dependent manner. Such use-dependent activity then allows AMPAkines to enhance endogenous functions of AMPA receptors in specific brain regions. Recent data suggest that AMPAkines can inhibit acute and chronic pain by specifically increasing postsynaptic glutamatergic signaling in the NAc, and hence these drugs have the potential to modulate the PL-NAc circuit to treat pain [39, 40]. However, it is not clear if AMPAkine potentiation of the postsynaptic function in the NAc can fully activate the corticostriatal pathway to treat pain.

In this study, we examined the role of the PL-NAc projection in pain regulation, using acute rodent pain models as well as models of chronic inflammatory and neuropathic pain. We found that optogenetic activation of the PL provided significant anti-nociceptive effects, as did direct AMPAkine activation of the NAc. Interestingly, AMPAkine potentiation of the glutamatergic signaling in the NAc partially reconstituted the effect of optogenetic activation of the PL on nocifensive withdrawals, but it fully occluded the effect of PL activation on reducing pain aversion. These results indicate that the NAc is an important target for the PL in pain regulation, especially in the regulation of the affective component of pain, and that AMPAkines can strongly modulate the corticostriatal circuit to treat acute and chronic pain.

## Results

### AMPAkine treatment in the NAc core partially reconstitutes the anti-nociceptive effects of PL

**Fig. 1:**
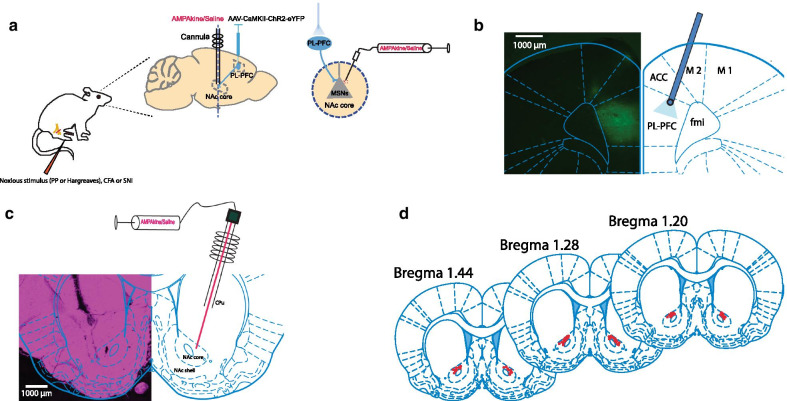
Experimental design and location of intracranial viral injections and cannula placements. **a** Schematic for in vivo optogenetic targeting of the PL and AMPAkine treatment in the NAc. **b** Histologic expression of Channelrhodopsin (ChR2) in the PL. **c** Representative brain slice showing the intracranial infusion site in the NAc core. **d** Schematic showing tip of injectors in the NAc core

Previous studies have demonstrated that the PL-NAc core projection strongly modulates acute pain behaviors [6–9]. Here we sought to examine the specific effect of AMPAkine potentiation of the postsynaptic function of NAc neurons for pain control in the corticostraital pathway. We used a calcium calmodulin-dependent protein kinase II (CaMKII) promotor to express light sensitive channelrhodopsin-2 (ChR2) and used light to activate the pyramidal neurons of the PL. We also infused CX546, a well-studied AMPAkine agent, to potentiate glutamate signaling via cannulas in the NAc core (Fig. 1a–d). CX546 (0.5 μl per side) was infused to the NAc core 15 min prior to behavior testing. We then compared the effect of PL activation with AMPAkine potentiation of the NAc.

**Fig. 2:**
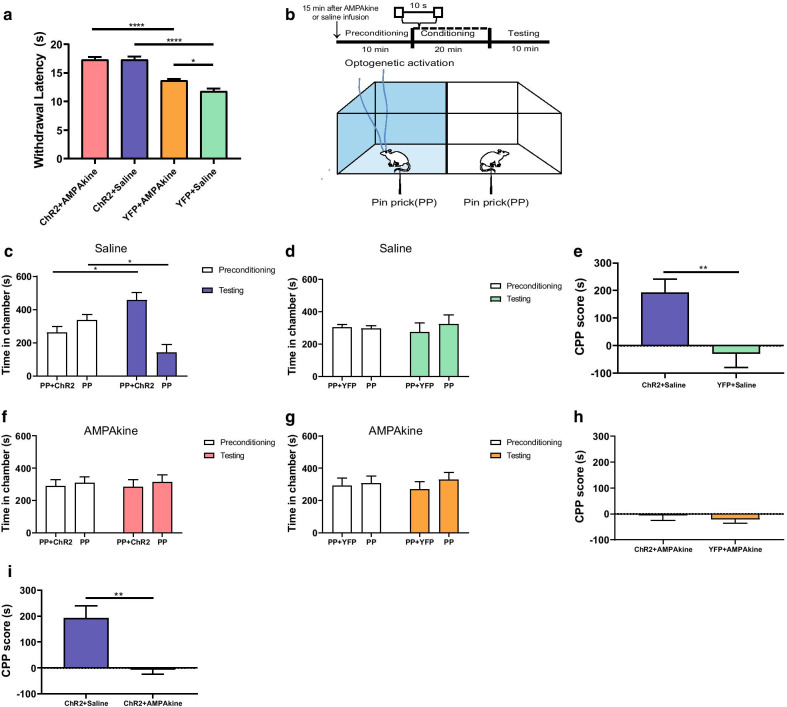
AMPAkine potentiation of the NAc partially reconstitutes the anti-nociceptive effects of PL**. a** Optogenetic activation of the PL has a greater impact than AMPAkine infusions into the NAc core on reducing sensory withdrawals on Hargreaves test. ChR2 group, n = 7; YFP group, n = 7; YFP + AMPAkine vs YFP + saline (*p = 0.0114); ChR2 + AMPAkine vs ChR2 + saline (*p* > 0.9999); ChR2 + AMPAkine vs YFP + AMPAkine (****p < 0.0001), ChR2 + saline vs YFP + saline (****p < 0.0001); Two-way ANOVA with repeated measures and Bonferroni’s multiple pair-wise comparisons. **b** Schematic of the CPP assay with optogenetic activation of the PL in the presence of noxious pin prick (PP) after AMPAkine or saline infusion into the NAc core. AMPAkine or saline was infused into the NAc 15 min prior to the CPP assay. During the CPP assay, one of the chambers was paired with optogenetic activation of the PL and PP; the other chamber was paired with PP alone. During the preconditioning or testing phase, no stimuli were given and rats were allowed free movement. **c** Control (saline-infused) rats, when presented with noxious stimuli (PP), preferred the chamber associated with optogenetic PL activation. n = 6, *p = 0.0101, paired Student’s *t*-test. **d** Control rats that had YFP injection did not demonstrate a preference for either chamber. n = 6, *p* = 0.5973, paired Student’s *t*-test. **e** CPP score for PL activation in the presence of noxious mechanical stimuli after saline infusion into the NAc core. n = 6, ^**^ **p** = 0.0098, unpaired Student’s *t* test. **f** Rats that received AMPAkine infusion prior to the CPP test, when presented with PP, did not demonstrate preference or aversion for the chamber associated with optogenetic PL activation. n = 6, *p* = 0.7524, paired Student’s *t*-test. **g** AMPAkine-infused rats that received YFP injection also demonstrated no preference or aversion for either chamber. n = 6, *p* = 0.1990, paired Student’s *t*-test. **h** After AMPAkine infusion into NAc core, the CPP score for PL activation was not increased compared with YFP control. n = 6, *p* = 0.5238, unpaired Student’s *t* test. **i** CPP scores indicate that AMPAkine treatment in the NAc eliminated the preference of chamber associated with PL activation. n = 6, **p = 0.0031, unpaired Student’s *t* test

First, we performed the Hargreaves test to assess the impact of PL or NAc activation on acute thermal pain regulation. Compared with saline control, we found that local infusion of CX546 prolonged the latency to paw withdrawal in response to noxious thermal stimuli in control rats (YFP + AMPAkine vs. YFP + saline, Fig. 2a). Interestingly, optogenetic PL activation occluded the anti-nociceptive effect of AMPAkine in the NAc (ChR2 + AMPAkine vs. ChR2 + saline, Fig. 2a). Furthermore, quantitatively, PL activation had a greater impact on sensory withdrawal than AMPAkine treatment in the NAc (ChR2 + AMPAkine vs. YFP + AMPAkine, Fig. 2a). These results indicate that potentiation of the AMPA receptor function in the NAc partially reconstitutes the anti-nociceptive effects of PL activation.

Pain consists of sensory and affective components. We used a classic two-chamber conditioned place preference assay (CPP) to assess the aversive component of pain [7, 9, 41–45]. AMPAkine (CX546) or saline was infused into the NAc core in the same rats prior to the CPP. After preconditioning in a two-chamber environment, rats received noxious mechanical stimulations in the form of pin pricks (PP) in both chambers. We used a 27-gauge syringe (Becton, Dickinson and Company, US) to deliver PP as noxious stimuli to rats' plantar region, and each PP stimulation was terminated by paw withdrawal. Noxious stimuli were repeated at 10 s intervals during the conditioning phase. During conditioning, one of the chambers was paired with laser treatment to optogenetically activate the PL at 20 Hz, whereas the other chamber was not paired with laser treatment. Finally, during the testing phase, rats were given free access to both chambers again with neither light modulation nor peripheral stimulations (Fig. 2b).

As expected, during the testing phase, saline-infused (control) rats preferred the chamber that was associated with optogenetic activation of the PL (Fig. 2c). In contrast, rats that expressed YFP and hence could not respond to light treatment did not display any preference for either chamber (Fig. 2d). The preference to PL activation could be further quantified by a CPP score, which was calculated by subtracting the amount of time spent in the chamber paired with light treatment during the preconditioning phase from the amount of time spent in that chamber during the test phase [44, 45]. A higher CPP score indicates preference for PL activation, suggesting that such activation reduced pain aversion. After saline (control) infusion into their NAc, rats clearly demonstrated a preference for PL activation in the presence of repeated noxious stimulations, indicating that PL activation reduces pain aversion (Fig. 2e).

To assess the role of AMPA receptor potentiation in the NAc in the regulation of pain aversion, we then performed the same test after AMPAkine (CX546) infusion in the NAc. Interestingly, we found that this time, rats that expressed ChR2 did not demonstrate a preference for the chamber associated with optogenetic activation of the PL, when they received PP stimuli in both chambers (Fig. 2f). Similarly, rats that expressed YFP (control) also did not display any chamber preference (Fig. 2g). Thus, after AMPAkine treatment in the NAc, rats failed to demonstrate further relief of pain aversion from PL activation (Fig. 2h). Indeed, when we compared the saline group with the AMPAkine group, we found that AMPAkine eliminated the increased CPP score induced by PL activation seen in control conditions (Fig. 2i). These results indicate that potentiation of postsynaptic glutamate signaling in the NAc may be able to achieve a similar regulatory effect on pain aversion as presynaptic PL activation.

### AMPAkine treatment partially reconstitutes the anti-nociceptive effects of PL in a model of chronic inflammatory pain

**Fig. 3:**
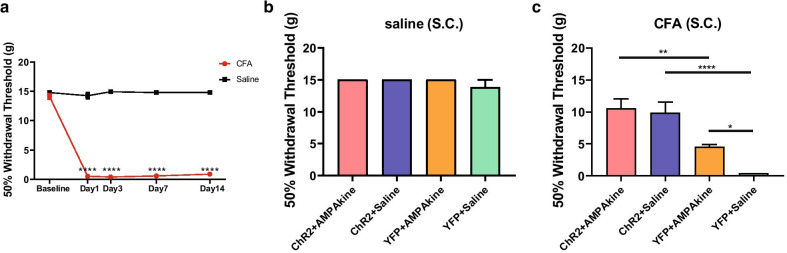
AMPAkine in the NAc partially reconstitutes the anti-nociceptive effects of PL on chronic inflammatory pain. **a** CFA treatment induces mechanical allodynia, compared with saline-treated rats. CFA group, n = 12; Saline group, n = 13; ****p < 0.0001, Two-way ANOVA with repeated measures and Bonferroni’s multiple pair-wise comparisons. **b** AMPAkine infusion into the NAc core and activation of PL did not change mechanical hypersensitivity in control rats. ChR2 group, n = 7; YFP group, n = 6; ChR2 vs YFP, after AMPAkine infusion (*p* > 0.9999), after saline infusion (*p* = 0.2760); AMPAkine vs saline, in ChR2 rats (*p* > 0.9999), in YFP rats (*p* = 0.3044); Two-way ANOVA with repeated measures and Bonferroni’s multiple pair-wise comparisons. **c** Optogenetic activation of the PL has greater impact than AMPAkine potentiation of the NAc core on the reduction of sensory withdrawal. ChR2 group, n = 6; YFP group, n = 6; ChR2 vs YFP, after AMPAkine infusion (**p = 0.0025), after saline infusion (****p < 0.0001); AMPAkine vs saline, in ChR2 rats (*p* > 0.9999), in YFP rats (*p = 0.0340); Two-way ANOVA with repeated measures and Bonferroni’s multiple pair-wise comparisons

Next we assessed the role of AMPAkines in the regulation of chronic pain using a well-known inflammatory pain model. We injected Complete Freund’s Adjuvant (CFA) subcutaneously into the hind paws of rats [44–46]. As expected, compared with saline-injected rats (control), CFA-treated rats displayed mechanical allodynia (Fig. 3a). 7 days after the CFA or saline injections, we measured mechanical allodynia during optogenetic PL activation, 15 min after saline or AMPAkine (CX546) infusion into the NAc (Fig. 3b, c). As expected, neither AMPAkine infusion, nor PL activation, had any impact on sensory withdrawals in control rats without chronic pain (Fig. 3b). In CFA-treated rats, however, we found that optogenetic activation of the PL reversed mechanical allodynia (ChR2 + saline vs. YFP + saline, Fig. 3c). However, the addition of AMPAkine in the NAc did not provide further anti-nociceptive effects (ChR2 + AMPAkine vs. ChR2 + saline, Fig. 3c). In contrast, AMPAkine treatment alone only produced approximately half of the anti-nociceptive effect of PL activation (YFP + AMPAkine vs. YFP + saline, ChR2 + AMPAkine vs. YFP + AMPAkine, Fig. 3c), whereas AMPAkine combined with PL activation produced similar anti-nociceptive effects as PL activation alone (ChR2 + AMPAkine vs. ChR2 + saline, Fig. 3c). These results indicate that potentiation of the AMPA receptor function in the NAc partially reconstitutes the anti-nociceptive effects of PL activation in the chronic pain state.

**Fig. 4:**
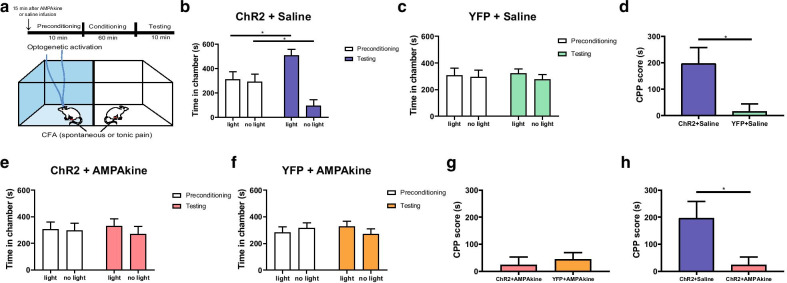
AMPAkine in the NAc core occludes the anti-aversive effect of PL on chronic inflammatory pain. **a** Schematic of the CPP test for tonic-aversive response in CFA-treated rats after AMPAkine or saline infusion in the NAc core. One of the chambers was paired with PL activation; the other chamber was not. No peripheral stimulus was given. **b** Saline-infused rats that received ChR2 injection preferred the chamber associated with PL activation. n = 6, *p = 0.0237, paired Student’s *t*-test. **c** Saline-infused rats that received YFP injection showed no preference for light treatment. n = 6, *p* = 0.5973, paired Student’s *t*-test. **d** CPP score for PL activation in the presence of spontaneous or tonic pain. n = 6, *p = 0.0232, unpaired Student’s *t* test. **e** AMPAkine pretreatment occluded the anti-aversive effects of PL activation. Rats which received CX546 infusion in the NAc core prior to CPP test did not demonstrate a preference for the chamber associated with PL activation. n = 6, *p* = 0.4405, paired Student’s *t*-test. **f** AMPAkine-infused rats which received YFP injection showed no chamber preference. n = 6, *p* = 0.1278, paired Student’s *t*-test. **g** AMPAkine pretreatment occluded the anti-aversive effects of PL activation in CFA-treated rats. After AMPAkine infusion into NAc core, the CPP score for PL activation was not increased compared with YFP control. n = 6, *p* = 0.6102, unpaired Student’s *t* test. **h** Compared with saline, AMPAkine treatment prior to CPP test occluded the anti-aversive effects of PL activation in CFA-treated rats. n = 6, *p = 0.0294, unpaired Student’s *t* test

In addition to peripheral hypersensitivity to evoked stimuli, chronic pain also causes spontaneous or tonic pain, manifested by aversive behavioral responses in the absence of obvious peripheral inputs [42, 47]. Recent studies have shown that the CPP assay can be used to confirm the presence of tonic pain aversion in animal models [6, 42, 48–50]. Thus, we performed the CPP assay in CFA-treated rats. We paired one of the chambers with optogenetic activation of the PL, and the opposite chamber without light treatment, during a long conditioning phase (Fig. 4a). We performed this CPP assay after AMPAkine or saline injection in the NAc core. Rats that received saline injections in the NAc, after a period of conditioning, preferred the chamber associated PL activation (Fig. 4b), indicating that the PL reduced tonic pain aversion. In contrast, YFP (control) rats did not demonstrate such preference (Fig. 4c). This tonic pain aversive response can be further quantified by a CPP score, which was calculated by subtracting the amount of time spent in the chamber paired with light treatment during the preconditioning phase from the amount of time spent in that chamber during the test phase [44, 45]. An analysis of the CPP score further indicates that PL activation is effective in removing tonic pain aversion (Fig. 4d).

Next, we examined the effect of PL activation on tonic pain aversion in rats that received AMPAkine treatment in the NAc (Fig. 4e-h). Here, CFA-treated rats that received ChR2 injection did not prefer light treatment, indicating that pre-treatment with CX546 occluded the effect of PL activation in reducing tonic pain aversion (Fig. 4e). Similarly, YFP-expressed rats also failed to display any chamber preference (Fig. 4f). When we compared the CPP scores, we found no difference in the CPP scores between ChR2 and YFP groups (Fig. 4g). Finally, when we compared saline and AMPAkine rats after CFA treatment, we found that the AMPAkine completely occluded increased CPP scores induced by PL activation (Fig. 4h).

### AMPAkine treatment partially reconstitutes the anti-nociceptive effects of PL in a model of chronic neuropathic pain

**Fig. 5:**
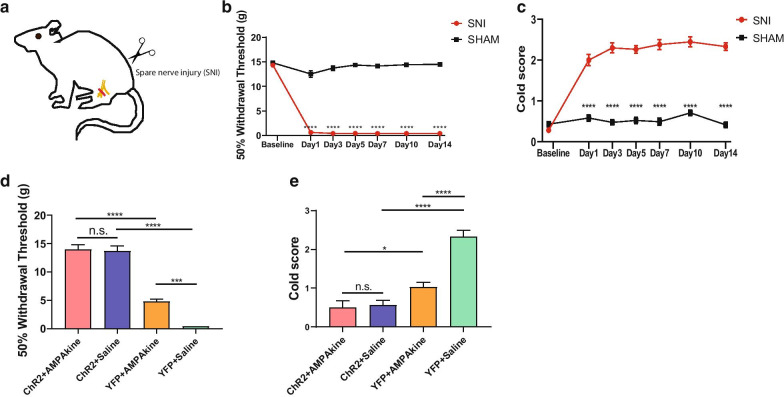
AMPAkine in the NAc partially reconstitutes the anti-nociceptive effects of PL on chronic neuropathic pain**. a** Schematic of the SNI model. **b** SNI treatment induces mechanical allodynia, compared with SHAM-treated rats. SNI group, n = 12; SHAM group, n = 13; ****p < 0.0001, Two-way ANOVA with repeated measures and Bonferroni’s multiple pair-wise comparisons. **c** SNI treatment induces cold allodynia, compared with SHAM-treated rats. SNI group, n = 12; SHAM group, n = 13; ****p < 0.0001, Two-way ANOVA with repeated measures and Bonferroni’s multiple pair-wise comparisons. **d** Optogenetic activation of the PL decreased mechanical allodynia in SNI-treated rats; AMPAkine infusion in the NAc decreased mechanical allodynia in YFP rats but not ChR2 rats. ChR2 group, n = 6; YFP group, n = 6; ChR2 vs YFP, after AMPAkine infusion (****p < 0.0001), after saline infusion (****p < 0.0001); AMPAkine vs saline, in ChR2 rats (*p* > 0.9999), in YFP rats (***p = 0.0002); Two-way ANOVA with repeated measures and Bonferroni’s multiple pair-wise comparisons. **e** Activation of the PL decreased cold allodynia in SNI-treated rats; AMPAkine infusion decreased cold allodynia in YFP but not ChR2 rats. ChR2 group, n = 6; YFP group, n = 6; ChR2 vs YFP, after AMPAkine infusion (*p = 0.0364), after saline infusion (****p < 0.0001); AMPAkine vs saline, in ChR2 rats (*p* > 0.9999), in YFP rats (****p < 0.0001); Two-way ANOVA with repeated measures and Bonferroni’s multiple pair-wise comparisons

To confirm our findings in the CFA model of inflammatory pain, we repeated the above experiments in a spared nerve injury (SNI) model chronic neuropathic pain [6, 35, 45, 51, 52]. Compared to sham surgery, SNI induced persistent mechanical and cold allodynia (Fig. 5a–c). 14 days after the SNI, we assessed the effect of PL activation on mechanical and cold allodynia after saline or AMPAkine infusion into the NAc core (Fig. 5d, e). We found that activating the PL reversed mechanical and cold allodynia (Fig. 5d, e). However, the addition of the AMPAkine in the NAc did not produce further anti-nociceptive effects. By itself, AMPAkine treatment produced approximately half of the anti-allodynic effect of PL activation, and combining AMPAkine activation of the NAc with PL activation produced similar anti-nociceptive effects as PL activation alone (Fig. 5d, e). These results are similar to what we found in the CFA model.

**Fig. 6:**
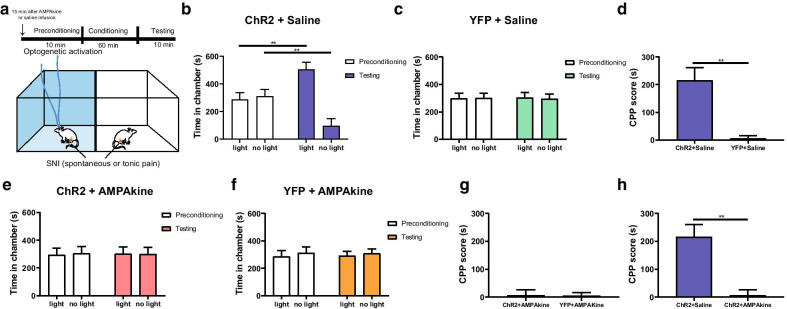
AMPAkine in the NAc core occludes the anti-aversive effect of PL activation on chronic neuropathic pain. **a** Schematic of the CPP test for tonic-aversive response in SNI-treated rats after AMPAkine or saline infusion in the NAc core. One of the chambers was paired with PL activation; the other chamber was not. No peripheral stimulus was given. **b** Saline-infused rats that received ChR2 injection preferred the chamber associated with PL activation. n = 6, **p = 0.0053, paired Student’s *t*-test. **c** Saline-infused rats that received YFP injection showed no preference for light treatment. n = 6, *p* = 0.5806, paired Student’s *t*-test. **d** CPP score for PL activation in the presence of spontaneous or tonic pain. n = 6, **p = 0.0012, unpaired Student’s *t* test. **e** AMPAkine pretreatment occluded the anti-aversive effects of PL activation. Rats which received CX546 infusion in the NAc core prior to CPP test did not demonstrate a preference for the chamber associated with PL activation. n = 6, *p* = 0.7514, paired Student’s *t*-test. **f** AMPAkine-infused rats that received YFP injection showed no chamber preference. n = 6, *p* = 0.6766, paired Student’s *t*-test. **g** AMPAkine pretreatment occluded the anti-aversive effects of PL activation in SNI-treated rats. After AMPAkine infusion into NAc core, the CPP score for PL activation was not increased compared with YFP control. n = 6, *p* = 0.9492, unpaired Student’s *t* test. **h** Compared with saline, AMPAkine treatment prior to CPP test occluded the anti-aversive effects of PL activation in SNI-treated rats. n = 6, **p = 0.0018, unpaired Student’s *t* test

Next, we used the CPP assay to assess the impact of AMPAkine treatment in the NAc on tonic pain in SNI-treated rats (Fig. 6a). We found that rats that received saline injections in the NAc, after a period of conditioning, preferred the chamber associated with PL activation (Fig. 6b). In contrast, YFP (control) rats did not demonstrate such preference (Fig. 6c, d). Next, we examined the effect of PL activation on tonic pain aversion in SNI-treated rats that received AMPAkine infusion in the NAc (Fig. 6e-h). These SNI-treated rats that expressed ChR2 did not prefer light treatment (Fig. 6e). Similarly, YFP-expressed rats also failed to display any chamber preference (Fig. 6f). When we compared the CPP scores, we found no difference in the CPP scores between ChR2 and YFP groups (Fig. 6g). Finally, when we compared saline and AMPAkine rats after SNI treatment, we found that pre-treatment with AMPAkine occluded the anti-aversive effect of PL activation (Fig. 6h). These data are compatible with findings from CFA-treated rats.

## Discussion

In this study, we have examined in detail the corticostriatal circuit in the regulation of acute and chronic pain. Specifically, we have studied the impact of AMPAkine treatment in the NAc. We found that AMPAkine potentiation of the postsynaptic AMPA receptors in the NAc completely occluded the anti-aversive effects of the PL, and partially reconstituted the effect of the PL on the regulation of nocifensive withdrawals.

In our study, we used AMPAkines to specifically stimulate the postsynaptic function of NAc. AMPA receptors are the main excitatory glutamate receptors in the nervous system [53]. AMPAkines are a class of synthetic agents that bind to an allosteric site on the AMPA receptor [37, 38]. The binding of AMPAkines slows the kinetics of AMPA receptor deactivation to enhance the inward excitatory synaptic current in a use-dependent manner [37, 38]. Thus, a key function of AMPAkines is to potentiate, rather than directly activate, postsynaptic signaling. AMPAkines have been shown to stimulate the respiratory drive by increasing excitatory inputs of neurons in the pre-Botzinger complex to treat opioid-induced hypoventilation [54–59]. AMPAkines have also been studied in depression, schizophrenia, Alzheimer’s disease, and Huntington’s disease [37, 60–63]. Furthermore, AMPAkines such as CX546 have been shown to prevent sedative-induced synaptic deficits in the brain [64]. Importantly, previous studies have shown that AMPAkines have anti-nociceptive properties in acute incisional and chronic pain conditions [39, 40, 65], and that potentiation of the postsynaptic function of the NAc appears to be crucial for these properties [39].

The key finding in our study is that CX546, a well-studied AMPAkine, fully occluded the anti-aversive function of PL neurons and partially reconstituted the effects of these neurons in controlling sensory withdrawals. Sensory and aversive components of pain are thought be regulated by different pathways [66, 67]. Whereas nocifensive withdrawals are mediated by spinal reflex, descending tracks originating from the cortex can strongly influence such responses [19, 68, 69]. The PL is part of a prefrontal network that is known to provide top-down regulation for sensory processes [70–73]. Studies have shown that activation of the output neurons from the PL can relieve pain, whereas the inhibition of these neurons have the opposite effects [5–8, 22, 74–76]. The PL has multiple descending projections. In addition to the NAc, PL projections to the amygdala and especially the periaqueductal gray are known to produce inhibitory effects on the spinal cord to modulate nocifensive withdrawals [5–8, 11, 22, 75, 77–79]. Thus, our results here are compatible with these previous findings and confirm that parallel descending pathways from the prefrontal cortex likely combine together to regulate the ascending transmission of the pain signal and to influence nocifensive responses.

The PL has a strong projection to the core subregion of the NAc as well as a weaker projection to the shell subregion [80–82]. The projections from the PL to the NAc have been well documented to play a key role in pain regulation [6, 7, 9], and depotentiation of these projections in the chronic pain state contributes to enhanced aversion [80]. The NAc is known as the hub for reward- and aversion-types of behaviors across species [23–28]. While the NAc may regulate sensory withdrawals [6, 33, 39], recent work has demonstrated that the activation of the NAc, in particular the NAc core, plays an especially pronounced role in regulating the aversive symptoms of pain [6, 35, 80, 83, 84]. AMPA potentiators such as AMPAkines have been shown to potentiate calcium permeable AMPA receptors in the NAc, and these receptors in turn are also known to endogenously regulate the affective component of pain in the NAc core [6, 35, 80, 85]. Thus, it is not surprising that AMPAkine potentiation of the NAc can have strong anti-aversive properties and occlude the effect of presynaptic activation of the PL. It should be noted, however, that our results do not rule out the possibility that AMPAkine activation of the NAc can also act outside the corticostriatal pathway to produce independent anti-aversive effects.

An alternative explanation for our results is that PL stimulation produced a ceiling effect. While allodynia tests are known to produce ceiling effects, such effects are less likely to be observed in place preference tests. Furthermore, previous studies have shown that the NAc is an important target for the PL in its regulation of pain, as inhibiting synaptic outputs from the PL to pyramidal neurons in the NAc blocked sensory and affective components of pain [6, 7, 9, 80], and that blocking AMPA receptors in the NAc core could enhance pain-associated affective changes [6, 35, 39]. Thus, it seems more likely that activation of the NAc mediates at least part of the pain-relieving effects of PL activation. At the same time, mechanical and cold allodynia measures latency and threshold to noxious inputs, whereas aversion produces a more cumulative measure of nociceptive responses over time. Thus, another possible explanation for our results is that the NAc plays a more important role in integrating the pain experience over time rather than setting thresholds for acute nociceptive responses, whereas the PL carries out both functions.

The present study targeted male rats. However, previous studies have shown significant sex difference in the cortical and subcortical processing of pain [86, 87]. Thus, future studies are needed to investigate potential sex differences in the analgesic effects of activation of NAc by AMPAkines.

In summary, our results show that AMPAkine activation of the NAc core partially reconstitutes the anti-nociceptive effect of PL activation and may play an even greater role in the anti-aversive effect of these neurons. These results indicate that the NAc is an important target for PL in its regulation of pain, and that AMPAkines may be important agents for the treatment of acute and chronic pain, with a particular role in the affective symptoms of pain.

## Methods and materials

### Animals

All animal care and experimental studies were performed according to the guidelines of the New York University School of Medicine (NYUSOM) Institutional Animal Care and Use Committee (IACUC) to ensure minimal animal use and discomfort, and were consistent with the NIH *Guide for the Care and Use of Laboratory Animals* (publication number 85–23). Male Sprague–Dawley rats were purchased from Taconic Farms (Albany, NY, USA) and kept at the vivarium facility in the NYU Langone Science Building, where is controlled humidity, temperature, and 12 h (6:30 AM to 6:30 PM) light–dark cycle. Food and water were available ad libitum. Animals weighed between 250 to 300 g arrived to the facility, and they were given 10–14 days on average to adjust to the new environment before the onset of any experiments.

### Virus construction and packaging

Recombinant AAV (adeno-associated virus) vectors were serotyped with AAV1 coat proteins, and then packaged at Addgene viral vector manufacturing facilities. Viral titers were approximately 5 × 10^12^ particles per milliliter (particles/ml) for AAV1.CAMKII.ChR2-eYFP.WPRE.hGH and AAV1.CAMKII(1.3).eYFP.WPRE.hGH.

### Drugs

CX546 (Tocris Bioscience, USA) was first suspended in dimethyl sulfoxide (DMSO) and then subsequently re-suspended in 0.9% saline (Hospira, USA) to a final concentration of 800 μM [39] for intra-NAc core infusions in naïve-, CFA-, SNI-treated rats. For intra-NAc core infusions, 0.5 μL of CX546 was locally infused to each side of the brain, while same volumes of 0.9% saline were applied in the control group. Rats were given at least 7 days to recover from cannula implantation before intracranial administrations and behavioral tests. All intra-NAc core infusions were performed 7 days after CFA (S.C.) injection or 14 days after SNI surgery. After the infusions, rats were given up to 15 min to rest before starting Hargreaves test, mechanical allodynia, cold allodynia, and two-chamber conditioned place preference assay (CPP).

### Intracranial viral injections and stereotaxic optic fiber and cannula implantation

As previously described [6, 35], rats were anesthetized with isoflurane (1.5%–2%). Virus as specified above was only delivered to the prelimbic PFC (PL) in all of the experiments. Briefly, rats were bilaterally injected manually with 0.6 μL of viral vectors at a slow rate of 0.1 μL every 20 s by using a 26 gauge 1 μL Hamilton syringe at AP (anteroposterior) + 2.9 mm, ML (mediolateral) ± 1.6 mm, and DV (dorsoventral) -3.7 mm with tips angled 12.5° toward the midline. After viral injection, the microinjection needles were then left in place for 10 min additionally, raised 1 mm, and further left for another additional 5 min before being slowly raised out of the brain, so as to allow for the diffusion of virus particles away from injector and to minimize spread of viral particles along the injection tract. Next, rats were bilaterally implanted with 200 μm optic fibers held in 1.25 mm ferrules (Thorlabs, Newton, NJ, USA) in the PL with coordinates: AP + 2.9 mm, ML ± 1.6 mm, DV − 3.2 mm, with tips 12.5° toward the midline. Optic fibers with ferrules were held in place by dental acrylic. The rats were given at least three weeks for virus to be expressed before behavioral tests.

For cannula implantations, as described previously [39, 88], rats were anesthetized with isoflurane (1.5–2%). Rats were stereotactically implanted with two 26 gauge guide cannulas (PlasticsOne Technologies, USA) bilaterally in the NAc core with the following coordinates at: AP + 1.3 mm, ML ± 2.9 mm, DV − 4.5 mm, with tips 8.5° toward the midline. Cannulas were held in place by dental acrylic and were kept clean and patent with occlusion stylets. Viral injection, fiber and cannula implantation were implemented in the same day.

### Intracranial pharmacology

For intracranial injections, solutions were loaded into two 30 cm lengths tubing of PE-50 and separately attached at one end to a 10 µL Hamilton’s syringe filled with distilled water, and at another end to a 33 gauge injector cannula, which extended 2 mm past the implanted guides. Bilateral delivery of 0.5 µL injection solution to each side took place over the course of 100 s and the injector cannulas were left in place for another 60 s to allow for diffusion of injectate solution into the brain after the injection was completely finished. Behavioral tests were done 15 min after intracranial injections. After behavioral experiments were completed, re-sterilized stylets were reinserted into the guides.

After animal sacrifice, cryogenic brain sections were collected with a thickness of 20 μm using a Microm HM525 Cryostat (Thermo Fisher Scientific, USA) and analyzed for the localization of cannula with histologic staining. Images were reviewed in a blinded fashion, and animals with improper cannula placement and occluded cannula guides (< 10%) were excluded from further analysis.

### Immunohistochemistry

Rats were deeply anesthetized with isoflurane and transcardially perfused with ice-cold PBS, and followed by ice-cold 4% PFA (paraformaldehyde) in PBS. Brains were fixated in PFA overnight and transferred to 30% sucrose in PBS to equilibrate for three days as previously described [6, 7, 9]. Following this, 20 µm coronal sections were collected by using Leica CM3050s cryostat (Leica Biosystems, Germany) and washed with PBS for 10 min. Sections were washed in PBS and coverslipped with Vectashield mounting medium. Sections were made after viral transfer for opsin verification, and these were stained with anti-rabbit GFP (1:500, #AB290, Abcam, USA). Secondary antibody was anti-rabbit immunoglobulin G (Ig G) conjugated to Alexa Fluor 488 (1:500, Life Technologies, USA). Images were acquired with a Zeiss LSM 700 Confocal Microscope (Carl Zeiss, Thornwood, NY, USA). Images containing cannulas were stained with cresyl violet and examined at 10 × magnification with an Axio Zoom widefield microscope (Carl Zeiss, Thornwood, NY, USA).

### Complete Freund’s adjuvant (CFA) administration

0.1 mL of CFA (Mycobacterium tuberculosis, Sigma-Aldrich, USA) was suspended in an oil saline (1:1) emulsion and then injected subcutaneously (S.C.) into the plantar aspect of the left hind paw for inducing chronic inflammatory pain [8, 45]. Control rats received an equal volume of saline injection subcutaneously into the plantar aspect of the left hind paw.

### Spared nerve injury (SNI) surgery

The procedure of SNI surgery was previously described in detail [6, 51, 52]. In brief, rats were anesthetized with isoflurane (1.5 to 2%) and the skin on the lateral surface of the right thigh of them was incised. A section was then opened through the bicep femoris muscle to expose the main sciatic nerve and its three terminal branches: common peroneal, tibial and sural nerves. The nerves of tibial and common peroneal were separately tied off with 5–0 nonabsorbent silk sutures at the proximal point of the trifurcation. Next, the nerves were resected distal to each knot, and approximately 5 mm of the distal ends were removed to prevent nerve reattachments. The sural nerve was left unharmed. In SHAM surgeries (control group), all three branches of the sciatic nerve were exposed, but not tied and cut. The muscle layer was sutured closed with 4–0 absorbable sutures and the skin with 3–0 silk sutures.

### Animal behavioral tests

Prior to behavioral tests, and 15 min after bilateral intracranial administrations, optic fibers were connected to a 473 nm laser diode with an FC/PC adapter (Shanghai Dream Lasers, China). And the laser intensity was measured with an instrument of power meter (Thorlabs, Newton, NJ, USA). The output of laser was delivered using a TTL pulse-generating box (Tucker-Davis Technologies, USA), and then was split evenly to two fibers (for bilateral stimulation) with a splitter. Before behavioral tests, laser output in each terminal of the two fibers was verified with an instrument of power meter to ensure that equal power was provided. As previously described [6, 7], a laser protocol that included alternating light-on and light-off epochs for 30 s each was provided for the duration of Hargreaves test, mechanical allodynia test, cold allodynia test, and CPP test. Light was delivered at 20 Hz with 10 ms pulse length within the light-on epoch.

### Hargreaves test (Plantar test)

The Hargreaves test was performed to evaluate acute thermal pain [89, 90]. For measuring the latency of paw withdrawal, we used a mobile radiant heat-emitting equipment with an aperture of 10 mm in diameter (37,370-Plantar Test, Ugo Basile, Italy) to produce acute noxious thermal stimuli. Rats were placed individually in a clear plexiglass chamber over a glass table and left to acclimate before the onset of testing. The mobile heat generator was aimed at the plantar surface of the rat’s left hind paw, and then an infrared (IR) intensity of 40 was used to provide acute noxious stimulation. The latency of paw withdrawal was recorded automatically, and IR stimuli were terminated by paw withdrawals or held for a maximum of 30 s. Paw withdrawals resulting from weight shifting or locomotion were not counted and the trials were repeated. 15 min after AMPAkine or saline infusion into NAc core, separate trials were conducted with optogenetic activation in ChR2 or YFP expression rats. The measurements were at least repeated five times at 5 min intervals on the left hind paw, and the averages of the five measurements for each trial were taken and further analyzed.

### Mechanical allodynia test

A traditional Dixon up-down method with von Frey filaments was used to measure mechanical allodynia [9, 52, 91, 92]. The rats were individually placed in clear plexiglass chambers on top of a mesh table and allowed to acclimate for 20 min prior to testing. 15 min after AMPAkine or saline infusion into NAc core, separate measurements were conducted with optogenetic activation in ChR2 or YFP expression rats. Von Frey filaments with logarithmically incremental stiffness (0.45, 0.75, 1.20, 2.55, 4.40, 6.10, 10.50, 15.10 g) were applied to the hind paw of the rats for measuring mechanical hypersensitivity, and a 50% withdrawal threshold was calculated using an up-down method [52]. For CFA or saline groups, filaments were applied vertically to the plantar surface of the left paw. And for SNI and SHAM groups, filaments were applied to the lateral one third of right hind paws (in the distribution of sural nerve) of rats with SNI or SHAM surgery.

### Cold allodynia test

Rats were individually placed in clear plexiglass chambers over a mesh table and left 20 min to acclimate. A drop of acetone was applied to the lateral plantar surface of the right hind paw (in the distribution of sural nerve). And as described previously [6, 93, 94], the following cold score of 0–3 was applied, 0: no visible response or brief lift of paw lasting < 0.5 s; 1: paw withdrawal lasting < 5 s; 2: extended withdraw of the paw lasting 5-10 s, with or without licking of the hind paw; 3: prolonged repetitive withdrawal of the hind paw lasting > 10 s. Acetone in total was applied five times to each paw, and an average cold score was calculated and further analyzed.

### Conditioned place preference (CPP) assay

Conditioned place preference (CPP) experiments were conducted in a standard two-compartment device, consisting of two compartments of equal size connected with an opening large enough for rats to move through freely, as described previously [44, 45]. The CPP protocol consists of preconditioning (baseline), conditioning, and testing phases. The preconditioning phase was 10 min, and rats were allowed to travel through freely between the two chambers during this period. Animals spending more than 500 s or less than 100 s of the total time in each chamber during the preconditioning phase were not used in further analysis. Immediately following the preconditioning phase, the rats underwent the conditioning phase. For testing evoked pain induced by peripheral stimulation (pin prick, or PP), the conditioning phase of CPP was 20 min. We used a 27-gauge syringe (Becton, Dickinson and Company, US) to deliver PP as noxious stimuli to rats' plantar region, and each pin prick stimulation was terminated by paw withdrawal. One of the two chambers was paired with both peripheral stimulation (PP) and 20 Hz optogenetic stimulation, and the other chamber was only paired with peripheral stimulation (PP). The peripheral stimulus was repeated at 10 s intervals. Optogenetic activation and chamber pairings was counterbalanced during conditioning. Preconditioning, conditioning, and testing phases were conducted on the same day [44, 45]. For testing spontaneous or tonic pain induced by CFA or SNI, the conditioning phase of CPP was 60 min. One of the two chambers was paired with 20 Hz optogenetic stimulation, and the other chamber was not. Optogenetic activation and chamber pairings was also counterbalanced during conditioning. During the testing phase, the rats did not receive any peripheral stimulation or optogenetic activation and had free access to move between the two chambers for 10 min. Animal movements in each chamber during the whole CPP procedure were recorded by a camera with high-speed and high-resolution from above the apparatus and analyzed with the AnyMaze Version 6.32 software (Stoelting Co., Wood Dale, IL, USA). Increased time spent in a chamber during the testing phase as compared with the baseline indicated preference for that chamber. The CPP score was computed by subtracting the amount of time spent in the chamber paired with optogenetic stimulation during the preconditioning phase from the amount of time spent in that chamber during the test phase.

### Statistics

The results of behavioral experiments were given as mean ± SEM. To compare withdrawal latency in Hargreaves test for naïve rats, mechanical allodynia withdrawal thresholds for CFA-treated, SNI-treated and related control rats, and cold allodynia score for SNI-treated and related control rats, two-way ANOVA with repeated-measures and post hoc multiple pair-wise comparison Bonferroni tests were used. For the CPP assay, a paired Student’s *t* test was used to compare differences in the time spent in each treatment chamber before (preconditioning phase) and after conditioning (testing phase). A two-tailed unpaired Student’s *t*-test was used to compare differences in CPP scores under various testing conditions. For all tests in this study, a *p* value < 0.05 was considered statistically significant. All data were analyzed using GraphPad Prism Version 8.2.0 software (GraphPad, La Jolla, CA, USA).

## Data Availability

All the data and code are available from the corresponding author on reasonable request.

## Abbreviations

PFC: Prefrontal cortex
PL: Prelimbic prefrontal cortex
NAc: Nucleus accumbens
AMPA: α-Amino-3-hydroxy-5-methyl-4-isoxazolepropionic acid
MSNs: Medium spiny neurons
PAG: Periaqueductal grayto
RVM: Rostral ventral medulla
PP: Pin prick
CPP: Conditioned place preference assay
CFA: Complete Freund’s Adjuvant
SNI: Spared nerve injury
